# Evaluating Multi-Ancestry Genome-Wide Association Methods: Statistical Power, Population Structure, and Practical Implications

**DOI:** 10.1101/2025.03.11.25323772

**Published:** 2025-03-12

**Authors:** Julie-Alexia Dias, Tony Chen, Hua Xing, Xiaoyu Wang, Alex A. Rodriguez, Ravi K. Madduri, Peter Kraft, Haoyu Zhang

**Affiliations:** 1. Department of Biostatistics, Harvard T.H. Chan School of Public Health, Boston, MA, USA; 2. Division of Cancer Epidemiology & Genetics, National Cancer Institute, National Instituters of Health, Bethesda, MD, USA; 3. Cancer Genomics Research Laboratory, Frederick National Laboratory for Cancer Research, Leidos Biomedical Research Inc, Rockville, MD, USA; 4. Data Science and Learning division, Argonne National Laboratory, Lemont, IL, USA

**Keywords:** Genome-wide association studies, Population Stratification, Meta-analysis, UK Biobank, All of Us

## Abstract

The increasing availability of diverse biobanks has enabled multi-ancestry genome-wide association studies (GWAS), enhancing the discovery of genetic variants across traits and diseases. However, the choice of an optimal method remains debated due to challenges in statistical power differences across ancestral groups and approaches to account for population structure. Two primary strategies exist: (1) Pooled analysis, which combines individuals from all genetic backgrounds into a single dataset while adjusting for population stratification using principal components, increasing the sample size and statistical power but requiring careful control of population stratification. (2) Meta-analysis, which performs ancestry-group-specific GWAS and subsequently combines summary statistics, potentially capturing fine-scale population structure, but facing limitations in handling admixed individuals. Using large-scale simulations with varying sample sizes and ancestry compositions, we compare these methods alongside real data analyses of eight continuous and five binary traits from the UK Biobank (N≈324,000) and All of Us Research Program (N≈207,000). Our results demonstrate that pooled analysis generally exhibits better statistical power while effectively adjusting for population stratification. We further present a theoretical framework linking power differences to allele frequency variations across populations. These findings, validated across both biobanks, highlight pooled analysis as a robust and scalable strategy for multi-ancestry GWAS, improving genetic discovery while maintaining rigorous population structure control.

## Introduction

Genome-wide association studies (GWAS) have played a crucial role in identifying genetic variants associated with various traits and diseases^1,2^. However, these studies have historically been dominated by individuals of European ancestry, who comprised approximately 94.5% of study participants as of 2025^3^, limiting the broader applicability of genetic discoveries. This imbalance poses challenges for the generalizability of genetic findings across populations, since allele frequencies, linkage disequilibrium (LD) patterns, and genetic architectures vary across ancestries^4–6^. To address this gap, researchers have increasingly incorporated participants from diverse genetic backgrounds into multi-ancestry GWAS^7–11^. These studies leverage genetic diversity to identify novel variants and refine polygenic risk scores (PRS), with recent multi-ancestry PRS methods demonstrating improved cross-population risk prediction^12–17^.

Despite these advances, questions remain regarding the optimal methodology for multi-ancestry GWAS. Two primary strategies are commonly used to analyze multi-ancestry GWAS: pooled analysis and meta-analysis and. pooled analysis combines all participants, regardless of ancestry, into a single analysis, adjusting for population stratification using principal components (PCs) in the pooled sample. This method increases the total sample size, enables the inclusion of admixed individuals, and often leads to improved statistical power. However, it raises concerns about inflated false positives if population stratification is not fully accounted for, and may be less effective at capturing subtle local ancestry effects compared to ancestry-group-specific analyses.

Meta-analysis, in contrast, conducts ancestry-group-specific GWAS and then combines the summary statistics^18^. This method better accounts for fine-scale population structure, facilitates data sharing when individual-level data are restricted, and may better account for heterogenous effect sizes across populations. An extension, MR-MEGA^6^, leverages allele frequency differences among contributing studies to boost power and handle admixed individuals. However, this method introduces additional parameters that can reduce power, especially when dealing with complex admixture.

Both strategies can be implemented using fixed-effect or mixed-effect models. Fixed-effect modelling (e.g., PLINK2^19^), assumes genetic effects are constant across individuals, providing computational efficiency, but limited ability to handle cryptic relatedness. In contrast, mixed-effect modelling^20–24^ includes both fixed and random effects to account for population structure and relatedness, enhancing robustness at the cost of increased computational demands. This approach is particularly useful in large biobank studies, where cryptic relatedness is common, and case-control imbalances may introduce biases if not properly accounted for.

In this study, we systematically compare three methods: pooled analysis, fixed-effect meta-analysis, and MR-MEGA^6^, using both fixed-effect and mixed-effect frameworks. Our primary goal is to identify which approach optimally balances statistical power and population stratification control in multi-ancestry contexts. We conduct large-scale simulations with individuals from five ancestry groups, varying sample sizes, ancestry-group proportions, and outcomes (continuous and binary). To further assess the impact of varying levels of admixture, we simulate admixed individuals using the Admix-kit pipeline^25^. Finally, we validate our findings in real-world data by analyzing eight continuous and five binary traits from two large biobanks, the All of Us Research Program^26^ (AoU, N≈207,000) and the UK Biobank^27^ (UKB, N≈324,000).

Our results demonstrate that pooled analysis achieves higher statistical power than meta-analysis and MR-MEGA across a range of study designs while maintaining well-controlled type I error in realistic scenarios. We further propose a theoretical framework linking these power gains to allele frequency differences across ancestry groups. These findings support pooled analysis as a robust and scalable approach for multi-ancestry GWAS, improving genetic discovery and enhancing the generalizability of GWAS findings across populations.

## Material and Methods

### Theoretical Motivation for Method Discrepancies

Consider a multi-ancestry cohort comprising J distinct subcohorts (ancestry groups). Let $n_{j}$ denote the number of subjects in subcohort j, and let $f_{j}$ be the allele frequency of a causal variant in subcohort j. We assume that the allelic effect of this variant (β) is constant across ancestry groups. For individual i in population j, the genotype $g_{ij}$ follows a binomial distribution $g_{ij}\sim Bin\left(2,f_{j}\right)$. The phenotype is defined as

$$y_{ij}=\beta g_{ij}+\varepsilon_{ij},\;\varepsilon_{ij}\sim N\left(0,\sigma^{2}\right),$$

where $i=1,\ldots ,n_{j}$ and j=1,…,J, and $\sigma^{2}$ represents the residual variance.

#### Within-Population GWAS

If we conduct a separate linear regression in each subcohort (with intercept), we obtain

$${\overset{ˆ}{\beta }}_{j}=\frac{\sum_{i=1}^{n_{j}}\left(y_{ij}-{\overset{‾}{y}}_{j}\right)\left(g_{ij}-{\overset{‾}{g}}_{j}\right)}{\sum_{i=1}^{n_{j}}{\left(g_{ij}-{\overset{‾}{g}}_{j}\right)}^{2}},$$

and

$$Var\left({\overset{ˆ}{\beta }}_{j}\right)=\frac{\sigma^{2}}{\sum_{i=1}^{n_{j}}{\left(g_{ij}-{\overset{‾}{g}}_{j}\right)}^{2}},$$

where ${\overset{‾}{y}}_{j}$ and ${\overset{‾}{g}}_{j}$ are the phenotypic and genotype means, respectively, in subcohort j.

#### Pooled Analysis GWAS

When pooling all individuals across the J subcohorts into a single GWAS, we have:

$${\overset{ˆ}{\beta }}_{P}=\frac{\sum_{j=1}^{J}\sum_{i=1}^{n_{j}}\left(y_{ij}-\overset{‾}{y}\right)\left(g_{ij}-\overset{‾}{g}\right)}{\sum_{j=1}^{J}\sum_{i=1}^{n_{j}}{\left(g_{ij}-\overset{‾}{g}\right)}^{2}},$$

and

$$Var\left({\overset{ˆ}{\beta }}_{P}\right)=\frac{\sigma^{2}}{\sum_{j=1}^{J}\sum_{i=1}^{n_{j}}{\left(g_{ij}-\overset{‾}{g}\right)}^{2}},$$

where $\overset{‾}{y}$ and $\overset{‾}{g}$ are the global phenotype and genotype means, respectively.

#### Fixed-effect Meta-analysis

Using inverse-variance weighting, the fixed-effect meta-analysis estimate is

$${\overset{ˆ}{\beta }}_{M}=\frac{\sum_{j=1}^{J}\sum_{i=1}^{n_{j}}\left(y_{ij}-{\overset{‾}{y}}_{j}\right)\left(g_{ij}-{\overset{‾}{g}}_{j}\right)}{\sum_{j=1}^{J}\sum_{i=1}^{n_{j}}{\left(g_{ij}-{\overset{‾}{g}}_{j}\right)}^{2}},$$

and

$$Var\left({\overset{ˆ}{\beta }}_{M}\right)=\frac{\sigma^{2}}{\sum_{j=1}^{J}\sum_{i=1}^{n_{j}}{\left(g_{ij}-{\overset{‾}{g}}_{j}\right)}^{2}}.$$

As $n_{j}\to \infty$, for all j, both ${\overset{ˆ}{\beta }}_{M}$ and ${\overset{ˆ}{\beta }}_{P}$ converge to β.

#### Asymptotic Relative Efficiency.

We define the asymptomatic relative efficiency (ARE) of meta-analysis vs. pooled-analysis as:

$$\text{ARE}\left({\overset{ˆ}{\beta }}_{P},{\overset{ˆ}{\beta }}_{M}\right)=\frac{Var\left({\hat{\beta }}_{M}\right)}{Var\left({\hat{\beta }}_{P}\right)}=\frac{\sum_{j=1}^{J}\sum_{i=1}^{n_{j}}{\left(g_{ij}-\overset{‾}{g}\right)}^{2}}{\sum_{j=1}^{J}\sum_{i=1}^{n_{j}}{\left(g_{ij}-{\overset{‾}{g}}_{j}\right)}^{2}}.$$

It follows that

$$\text{ARE}\left({\overset{ˆ}{\beta }}_{P},{\overset{ˆ}{\beta }}_{M}\right)=\frac{\sum_{j=1}^{J}\sum_{i=1}^{n_{j}}{\left(g_{ij}-{\overset{‾}{g}}_{j}\right)}^{2}+\sum_{j=1}^{J}n_{j}{\left({\overset{‾}{g}}_{j}-\overset{‾}{g}\right)}^{2}}{\sum_{j=1}^{J}\sum_{i=1}^{n_{j}}{\left(g_{ij}-{\overset{‾}{g}}_{j}\right)}^{2}}>1,$$

because of the standard ANOVA decomposition:

$$\sum_{j=1}^{J}\sum_{i=1}^{n_{j}}{\left(g_{ij}-\overset{‾}{g}\right)}^{2}=\sum_{j=1}^{J}\sum_{i=1}^{n_{j}}{\left(g_{ij}-{\overset{‾}{g}}_{j}\right)}^{2}+\sum_{j=1}^{J}n_{j}{\left({\overset{‾}{g}}_{j}-\overset{‾}{g}\right)}^{2}.$$

The term $\sum_{j=1}^{J}n_{j}{\left({\overset{‾}{g}}_{j}-\overset{‾}{g}\right)}^{2}$ represents the sample-size-weighted variance in allele frequencies across populations. When allele frequencies differ substantially across subcohorts, the pooled analysis benefits from leveraging higher variance in carrier counts across populations, improving power. If one population is disproportionately large (e.g. $n_{2}\ll n_{1}$ in a two-population settings), the advantage of pooled analysis diminishes because the dominant population drives the association signal, and the sample-size-weighted variance in allele frequencies will be small.

### Implementation of Methods

We applied the following approaches in both simulations and real data analyses using AoU and UKB datasets.

#### Pooled and Meta-analysis.

For pooled analysis, GWAS was performed on the entire dataset by combining all ancestry groups into a single analysis. Population structure was adjusted using top ten cross-ancestry-group PCs derived from the full pooled dataset, along with covariate adjustments for age and sex. Variants were included in the pooled analysis if they had MAF >1% in at least one ancestry group. For meta-analysis, GWAS was conducted separately within each ancestry group. Top ten ancestry-group-specific PCs were used for population structure adjustment, along with age and sex as covariates. Each ancestry-specific GWAS included only variants with MAF >1% within that ancestry group. Summary statistics from individual ancestry analyses were then combined using a fixed-effect meta-analysis approach with inverse-variance weighting. As a result, variants were included in the final meta-analysis if they met the MAF >1% threshold in at least one ancestry group, ensuring comparability with pooled analysis.

Both approaches used REGENIE^20^ (--qt for continuous traits and --bt for binary traits) for the mixed-effect modelling and PLINK2^19^ (--linear for continuous traits) for the fixed-effect modelling. REGENIE was selected as a scalable approach for mixed-model association testing, as it uses a local ridge regression framework to adjust for population structure and relatedness. While REGENIE does not explicitly introduce a random effect term, its regularization-based approach functionally approximates mixed-effect modelling by accounting for cryptic relatedness and polygenic effects, distinguishing it from standard fixed-effect models implemented in PLINK2.

#### MR-MEGA.

MR-MEGA extends meta-analysis by incorporating ancestry-group-specific summary statistics while explicitly accounting for allele frequency differences across ancestry groups. MR-MEGA models ancestry-group-specific genetic effects as a function of population structure, using PCs derived from allele frequency variation across groups. The input for MR-MEGA is similar to standard meta-analysis, using summary statistics from either mixed-effect or fixed-effect GWAS results. The number of axes of genetic variation used in MR-MEGA (--pc) was set to the maximum of the number of distinct genetic ancestry groups minus three. Specifically, for UKB samples, --pc was set to 2, while for AoU samples, --pc was set to 3. MR-MEGA was applied only when combining data from at least four ancestry groups. For example, MR-MEGA was not used for prostate cancer in UKB, as only three ancestry groups had a sufficient number of cases. It is important to note that: MR-MEGA restricts its output to variants with MAF > 1% in all genetic ancestry groups, while meta-analyses include variants with MAF > 1% in at least one ancestry group.

### Type I Error Simulations

To evaluate Type I error, we used real genomic data from six genetic ancestry groups as defined in AoU^26^: African (AFR), Admixed American (AMR), East Asian (EAS), European (EUR), Middle Eastern (MID) and South Asian (SAS), to simulate null phenotypes under varying degrees of population stratification. Employing actual genotype data allows us to capture realistic LD structures within each ancestry. For both ancestry-group-specific and cross-ancestry-group scales, stratification was introduced by varying the percentage of the null phenotype’s variance explained by the first ten PCs, ranging from 0% to 5%.

#### Percentage of Variance Explained by PCs.

Before simulating phenotypes, we estimated the variance explained by the first ten PCs in real polygenic traits. Five representative traits were selected: height, high-density lipoprotein cholesterol (HDL), low-density lipoprotein cholesterol (LDL), total cholesterol (TC), and waist circumference. To quantify the variance explained by ancestry-group-specific PCs and cross-ancestry-group PCs, we applied a two-step regression approach. First, within each ancestry group, we regressed out age and sex from the trait values and obtained the residuals. Next, we estimated the proportion of variance explained ($R^{2}$) for each PC set by comparing models with different covariate adjustments. The variance explained by ancestry-group-specific PCs was computed as the difference in $R^{2}$ between the full model, which included both cross-ancestry-group PCs and ancestry-group-specific PCs, and the reduced model, which included only cross-ancestry-group PCs. Similarly, the variance explained by cross-ancestry-group PCs was measured as the difference in $R^{2}$ between the full model, which included both PC sets, and the reduced model, which included only ancestry-group-specific PCs. Confidence intervals are obtained via bootstrapping using 10,000 replicates (Supplementary Table 1). These empirical estimates guided our selection of α values (0, 0.2%, 0.5%, 1%, 2%, 5%) in the subsequent simulations.

#### Null Phenotype Generation.

We simulated population stratification separately on ancestry-group-specific and cross-ancestry-group scales. Empirical results (Supplementary Table 1) indicated that after accounting for ancestry-group-specific PCs, cross-ancestry PCs explained 0% variance, justifying this independent modelling. To simulate ancestry-group-specific population stratification, we set

$$y_{ij}=\sum_{k=1}^{10}\delta_{jk}PC_{ijk}+\varepsilon_{ij},\;\varepsilon_{ij}\sim N(0,\sqrt{1-\alpha }),$$

where $PC_{ijk}$ is the $k^{\text{th}}$ PC for individual i in ancestry j. $\delta_{jk}=\sqrt{\frac{w_{jk}\alpha }{\left.Var({PC}_{jk}\right)}}$, is the effect for kth ancestry-group-specific PC in ancestry j,α is the total percentage of variance explained by the first ten ancestry-group-specific PCs, and $w_{jk}=\lambda_{jk}/\lambda_{j}$, with $\lambda_{jk}$ being the eigenvalue of the $k^{\text{th}}$ PC in ancestry j, and $\lambda_{j}$ the sum of the ten eigenvalues for ancestry j. This formulation of $\delta_{jk}$ ensures that population stratification effects are proportionally assigned across PCs while preserving phenotype variance. The resulting null phenotype has mean 0 and variance 1 within each ancestry group.

Similarly, to simulate cross-ancestry-group population stratification, we used

$$y_{ij}=\sum_{k=1}^{10}\delta_{k}PC_{ik}+\varepsilon_{ij},\;\varepsilon_{ij}\sim N(0,\sqrt{1-\alpha }),$$

where $PC_{ik}$ is the $k^{\text{th}}$ cross-ancestry-group PC for individual $i,\delta_{k}=\sqrt{\frac{w_{k}\alpha }{Var\left(PC_{k}\right)}}$, is the effect for kth cross-ancestry-group PC, α is the total percentage of variance captured by the top ten cross-ancestry-group PCs, and $w_{k}=\lambda_{k}/\lambda$, with $\lambda_{k}$ as the eigenvalue of the $k^{th}$ cross-ancestry-group PC, and λ as the sum of the eigenvalues of top ten cross-ancestry-group PCs. As in the ancestry-group-specific case, this design ensures that the null phenotype remains standardized with mean 0 and variance 1, while incorporating realistic population structure.

We simulated α∈(0,0.2%,0.5%,1%,2%,5%) to assess different degrees of population stratification. These scenarios reflect both observed values (e.g., 0% - 0.5%), and hypothetical extremes (e.g. 1% - 5%). This setup allowed us to assess whether each GWAS method (pooled, meta-analysis, MR-MEGA) controlled type I error appropriately in the presence of population structure on different scales.

### Statistical Power Simulations

#### Continuous Phenotypes.

We used a previously published simulated dataset of 600,000 independent subjects^13^ (120,000 from each of five ancestry groups: AFR, AMR, EAS, EUR, SAS), generated by HAPGEN2^28^ and the 1000 Genomes Project (1000G)^29^ as reference for LD and allele frequency structures (Supplementary Figure 1). To generate continuous phenotypes, we randomly selected 5% of variants from the HapMap3 variant list^30^ to be causal. Phenotypes for individual i in population j were simulated as:

$$Y_{ij}=\gamma_{j}+\sum_{l=1}^{L}X_{ijl}u_{jl}+\sum_{k=1}^{2}PC_{ijk}\delta_{jk}+\varepsilon_{ij},$$

where $\gamma_{j}\sim N(0,1)$ is an ancestry-group-specific intercept term. $X_{ijl}=G_{ijl}/\sqrt{2f_{jl}\left(1-f_{jl}\right)}$ is the standardized genetic value for variant l with mean 0 and variance 1. Under the assumption that variant effects on the standardized scale are constant across ancestry groups, we sampled causal effect sizes $u_{jl}\sim N(0,\frac{h^{2}}{L})$, with L being the number of causal variants, and the heritability $h^{2}$ being set to 0.4. This implies that the per-allele effect size varies with respect to the MAF in each group. Two ancestry-group-specific PCs were included, with $\delta_{jk}$ assigned based on the empirical estimates from UKB. The residual error variance was modeled as,

$$\varepsilon_{ij}\sim N\left(0,\sqrt{1-\left(h^{2}+\delta_{1k}^{2}var\left(PC_{1k}\right)+\delta_{2k}^{2}var\left(PC_{2k}\right)\right)}\right).$$

Ten replicates of continuous phenotypes were generated.

#### Binary Phenotypes.

To generate binary traits, we simulated a larger dataset of five million individuals (one million per ancestry group) using HAPGEN2 with 1000G as reference, to ensure adequate sample sizes for case-control sampling and accurate representation of allele frequency and LD structures. We randomly selected 1% causal variants across the genome. The binary outcomes were generated using a logistic model:

$$g\left(\text{Pr}\left(Y_{ij}=1\right)\right)=\alpha_{j}+\sum_{l=1}^{L}X_{ijl}u_{jl}+\sum_{k=1}^{2}PC_{ijk}\delta_{jk},$$

where $g(x)=\text{log}(\frac{x}{1-x})$, is the logit function. Similarly as continuous outcomes, we assume $u_{jl}\sim N(0,\frac{h^{2}}{L})$, where logit-scale genetic variance $h^{2}$ was set to 0.57 to mimic breast cancer^31^. The intercept term $\alpha_{j}$ was used to control disease prevalence. We assigned different $\alpha_{j}$ to mimic the estimated prevalence of breast cancer from SEER*Stat based on the past 28 years (Supplementary Table 2). After generating case-control status for all individuals, we randomly sampled cases and controls in a one-to-one ratio to create datasets with an average total sample size of 237,241 cases and 237,241 controls (detailed sample sizes by ancestry group described in Supplementary Table 3).

#### Admixed Individuals.

To evaluate admixture effects, we simulated an additional dataset of 240,000 individuals using Admix-kit^25^ and 1000G as the reference. The simulated dataset included four groups: 1. 60,000 individuals with 100% EUR ancestry 2. 60,000 individuals with 100% EUR ancestry 3. 60,000 subjects with 50% EUR and 50% AFR admixture 4. 60,000 subjects with 20% EUR and 80% AFR admixture (Supplementary Figure 2).

Admix-kit is a simulation tool that generates realistic admixed genomes by combining haplotypes from multiple ancestral populations. It models admixture as a generational process, where individuals inherit ancestry segments based on recombination patterns over multiple generations. The simulation was conducted in two steps. First, we expanded the reference haplotype data using HAPGEN2, generating a larger pool of phased haplotypes for EUR and AFR populations. To improve computational efficiency, we restricted the variants to those included in the HapMap3 variant list^30^. In the second step, we simulated admixture by drawing haplotypes from the expanded EUR and AFR datasets using the admix-simu function in Admix-kit. We modelled ten generations of admixture (--n-gen 10), allowing for recombination to shape local ancestry patterns. Ancestry proportions were assigned using the --admix-prop flag to reflect the desired admixture levels in each group. Continuous phenotypes for admixed individuals were simulated following the same methodology described in the previous section.

#### Statistical Power Evaluation.

We used two metrics to evaluate the performance of GWAS methods: 1. Exact recovery of causal variants, which measures the percentage of causal variants that are directly identified at genome-wide significance with $P<5\times 10^{-8}$. It is defined as the number of causal variants reaching genome-wide significance. 2. LD-based recovery of causal variants, which assesses the percentage of causal variants located within LD regions of genome-wide significant variants. Specifically, it is calculated as the number of causal variants with at least one genome-wide significant variant within a 500 kb region, divided by the total number of causal variants.

### Real Data Analysis in the All of Us Research Program and UK Biobank

For both AoU and UKB datasets, we analyzed eight continuous traits and five binary traits. The continuous traits included height, waist circumference, LDL, TC, HDL, calcium, creatinine, and estimated glomerular filtration rate (eGFR). The binary traits examined were asthma, coronary artery disease (CAD), type II diabetes (T2D), breast cancer (BC), and prostate cancer (PC). The corresponding concept IDs for AoU and Data Field IDs for UKB are provided in Supplementary Table 4. Subjects with measurements outside predefined ranges for each phenotype were excluded (Supplementary Table 4).

#### Data Processing and Sample Selection in AoU.

For AoU analyses, we utilized phenotype and genotype array data from AoU version 7.1. Participant information was collected in accordance with the AoU Research Program Operation Protocol. Detailed procedures related to genotyping, ancestry classification, quality control measures, and the exclusion of related participants are comprehensively documented in the AoU Research Genomic Research Data Quality Report. The AoU data includes both genotype array data and short-read whole genome sequencing (srWGS) data. Although genotype array data encompasses a larger sample size, ancestry and family relatedness information were only available for individuals with srWGS data. To ensure consistent ancestry classification, we restricted analyses to individuals with srWGS data, but we used genotype array variants for GWAS. Variants were filtered based on a MAF threshold of > 0.01 within any ancestry group, resulting in approximately 1.2 million variants.

We excluded flagged related subjects, subjects with ‘sex_at_birth’ missing (e.g., “Skip”, “I prefer not to answer”, “No matching concept”, “None”), and individuals with a predicted ancestry probability was ≤ 0.75. This threshold was applied to ensure a consistent comparison between pooled and meta-analysis approaches, as meta-analysis requires pre-defined ancestry groups. Including these individuals, who are likely admixed, would have been inconsistent with the ancestry-group-based framework used in meta-analysis. After these exclusions, the final dataset included 207,305 subjects across six genetic ancestry groups: 47,207 AFR, 36,500 AMR, 5,153 EAS, 115,701 EUR, 497 MID and 2,247 SAS (Supplementary Figure 3). Phenotype values were taken from the most recent available measurement, and age was calculated as the difference between the date of birth and the date of the latest phenotype measurement.

#### Data Processing and Sample Selection in UKB.

For UKB, we classified individuals into five major continental ancestry groups (AFR, AMR, EAS, EUR, SAS) using the 1000G^29^ as a reference. A random forest classification model was trained on the 1000G dataset, using the top 20 PCs as predictors and known ancestry group labels as outcomes. This trained model was then applied to the top 20 PCs within UKB to infer genetic ancestry. To minimize population structure confounding, we restricted analyses to unrelated individuals, resulting in a final dataset of 323,908 subjects across five genetic ancestry groups (6,864 AFR, 590 AMR, 586 EAS, 311,053 EUR and 5,734 SAS) (Supplementary Figure 4). Age in UKB was based on ‘age at visit’ (Data-Field 21003). Variants for UKB analyses were restricted to HapMap3^30^ + Multi-Ethnic Genotyping Arrays^32^ (MEGA) chip array, and further filtered using a MAF threshold of > 0.01 within at least one ancestry group, resulting in approximately 1.53 million variants.

#### PCA Computation.

To compute PCs, we pruned autosomal variants with MAF >1% across the pooled dataset, using an $r^{2}$ threshold of 0.1, a window size of 500kb with PLINK2^19^ (--indep-pairwise). This resulted in a set of 125,692 variants in AoU and 51,922 variants in UKB. The first 10 PCs were then computed separately within each genetic ancestry group and within the pooled dataset using PLINK2^19^ (--pca-approx).

## Results

**Figure 1:**
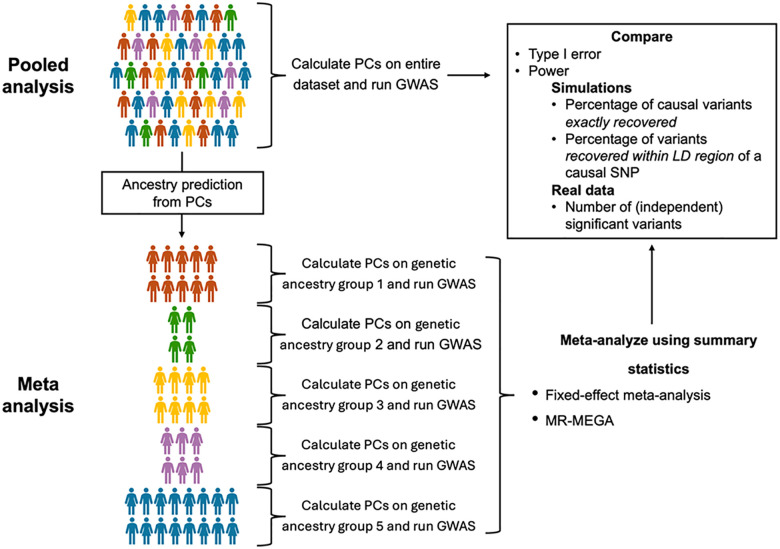
Overview of analysis. In the pooled analysis (top), principal components (PCs) are computed for the entire dataset, followed by a single GWAS. In the meta-analysis (bottom), PCs are calculated separately for each genetic ancestry group, and independent GWAS are conducted for each. Summary statistics are then combined through meta-analysis using inverse-variance weighting or MR-MEGA. The two approaches are compared based on type I error and statistical power. In simulations with causal variants statistical power is assessed by the percentage of causal variants exactly recovered and those within the 500kb linkage disequilibrium (LD) region of a causal variants. In real data analyses, statistical power is measured by the number of significant variants ($P\leq 5\times 10^{-8}$) and the number of independent signals (linkage disequilibrium $r^{2}<0.1$).

We evaluated three multi-ancestry GWAS strategies: meta-analysis, MR-MEGA^6^ and pooled analysis (Figure 1). Meta-analysis was implemented with standard fixed-effect meta-analysis using inverse variance weighting (Materials and Methods). All primary analyses employed mixed-effect modelling with REGENIE^20^ in the main text, while analyses using fixed-effect modelling was performed with PLINK2^19^ in the Supplementary Material. In pooled analysis, individuals from all ancestry groups were combined into a single dataset, with cross-ancestry-group PCs controlling for population stratification. In contrast, meta-analysis stratified individuals into genetically homogeneous ancestry groups before combining their summary statistics. MR-MEGA refined meta-analysis by explicitly modelling allele frequency differences among ancestry groups.

Our comparisons of multi-ancestry GWAS methodologies focused on two core metrics: 1. Type I error, evaluating whether each method properly controls false positives in the presence of population stratification 2. Statistical power, measured by (a) exact causal variant detection, which measures the proportion of causal variants reaching genome-wide significance, and (b) LD-based detection, which measures proportion of significant variants within 500 kb of a causal variant.

### Statistical Power Discrepancies Between Pooled and Meta-analysis

**Figure 2:**
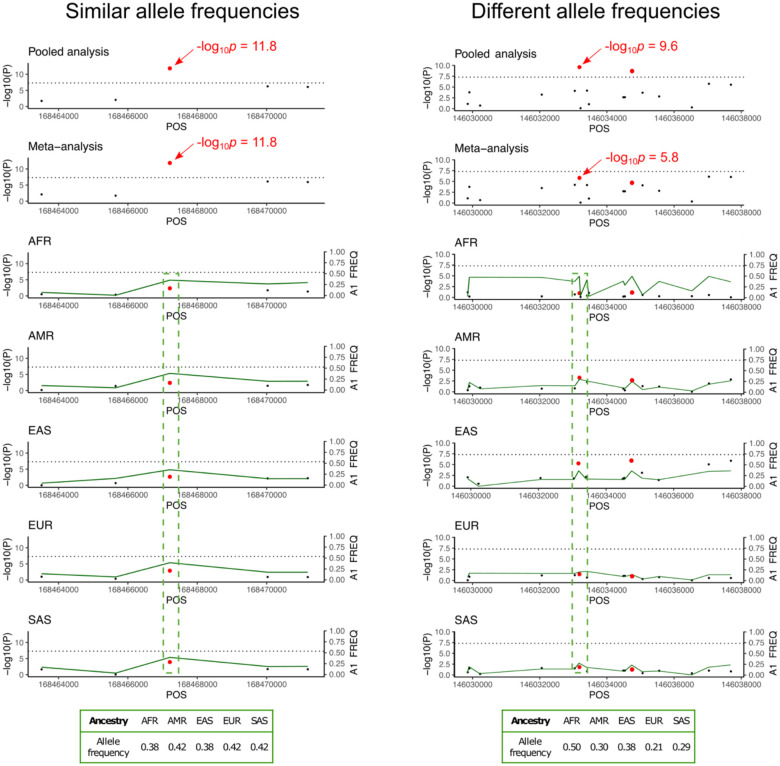
Illustration of pooled vs. meta-analysis approaches in multi-ancestry GWAS based on allele frequency differences. This figure demonstrates the theoretical differences in power between pooled and meta-analysis approaches in multi-ancestry GWAS are mainly driven by allele frequency differences. Manhattan plots show simulated results for two known causal variants under two scenarios: 1. Similar allele frequencies across ancestry groups (chromosome 4, rs11936438) result in equal statistical power for both pooled and meta-analysis ($-{\text{log}}_{10}\;p=11.8$, shown in left panel), 2. Divergent allele frequencies (chromosome 5, rs404167) show higher power in pooled analysis ($-{\text{log}}_{10}\;p=9.6$ compared to meta-analyses ($-{\text{log}}_{10}\;p=5.8$, shown in the right panel). The lower panels display ancestry-group-specific results for simulated ancestry groups: African (AFR), Admixed American (AMR), East Asian (EAS), European (EUR), and South Asian (SAS). The green line represents the minor allele frequency. The tables at the bottom summarize allele frequencies of the causal SNPs across ancestry groups, highlighting how different allele frequencies impact the statistical power of each approach.

Under the assumption of homogeneous allelic effects across ancestry groups, differences in statistical power between pooled and meta-analysis depend on allele frequency variance across populations (Figure 2). When MAFs are similar across populations, both methods yield comparable power. However, as MAF divergence increases, the relative advantage of pooled analysis becomes more pronounced (Materials and Methods). This advantage arises because pooled analysis integrates information across populations, allowing individuals from groups with high MAF to contribute more effectively to association detection. In contrast, meta-analysis treats each population separately before combining results, limiting the ability of high-MAF groups to enhance power in low-MAF populations. The power gain of pooled analysis is maximized when allele frequencies vary across ancestries and sample sizes are balanced. However, if one population is disproportionately large, the benefits of pooling diminish, as the dominant population drives association signals, reducing the influence of smaller ancestry groups. Overall, pooled analysis achieves better statistical power at a particular locus when allele frequencies differ across populations, with its advantage further amplified when highly divergent alleles are well-represented in larger sample sizes.

### Type I Error Simulations

We evaluated type I error by simulating null phenotypes across six populations using AoU data, ensuring that LD patterns and population stratification reflected real genomic architectures. We investigated both local population stratification, where the phenotype was associated with ancestry-group-specific PCs, and global population stratification, where the phenotype was associated with cross-ancestry-group PCs. To model varying degrees of stratification, we varied the proportion of the null phenotype’s variance explained by the first ten ancestry-group-specific and cross-ancestry-group PCs (Material and Methods).

**Figure 3:**
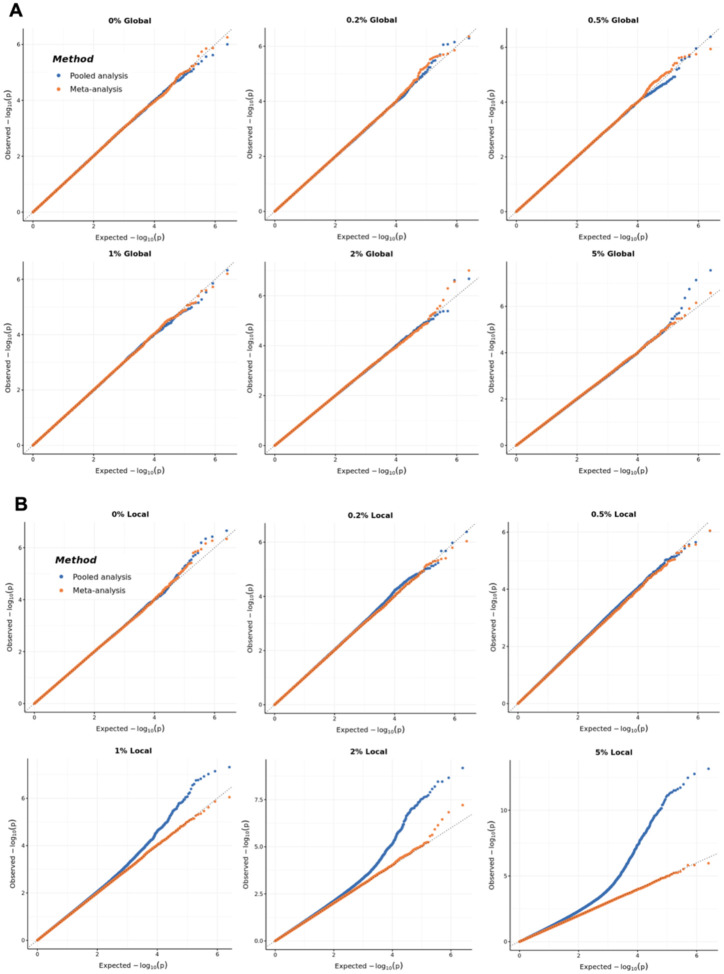
Quantile-Quantile (QQ) plots comparing the observed versus expected $-{log}_{10}(\text{p})$ values for meta-analysis (orange) and pooled analysis (blue) under varying levels of population structure. Null phenotypes were generated using the All of Us dataset, which includes individuals from six genetic ancestry groups: 47,207 African (AFR), 36,500 Admixed American (AMR), 5,153 East Asian (EAS), 115,701 European (EUR), 497 Middle Eastern (MID) and 2,247 South Asian (SAS) (see Methods). Panel A shows QQ plots of mixed-effect GWAS results for null phenotypes with varying percentages (0%−5%) of phenotypic variance are explained by the first ten cross-ancestry-group principal components (PCs). Panel B illustrate the same analysis ancestry-group-specific PCs, with similar ranges of variance explained. Divergence from the diagonal line indicates p-values inflation due to population structure in both local and global population stratification scenarios.

Under both fixed- and mixed-effect modelling approaches, type I error was well controlled with meta-analysis, regardless of the magnitude of ancestry-group-specific or cross-ancestry-group PC effects. Pooled analysis also controlled type I error when cross-ancestry-group PCs were associated with the outcome but exhibited inflation when more than 1% of the null phenotype’s variance was explained by ancestry-group-specific PCs. (Figure 3 for mixed-effect modelling; and Supplementary Figure 5 for fixed-effect modelling). However, real-world traits typically exhibit much lower ancestry-specific PC variance. In AoU, the median variance explained by the first ten ancestry-specific PCs is only 0.26% across the three largest ancestry groups (Supplementary Table 1). Higher ancestry-group-specific PC variance (>1%) was primarily observed in smaller ancestry groups such as EAS, SAS, and MID, where estimates may be less stable due to limited sample sizes. While large multi-ancestry GWAS may include some populations with small samples sizes, it is unlikely that most participating ancestry groups exhibit extreme PC variance exceeding 1%. Notably, our simulations only showed inflated p-values when multiple ancestries had PC variance above this threshold. This suggests that the level of ancestry-group-specific population structure required to induce meaningful bias in pooled analysis is substantially greater than what is typically observed in well-powered studies.

### Statistical Power Simulations

#### Continuous phenotypes

**Figure 4:**
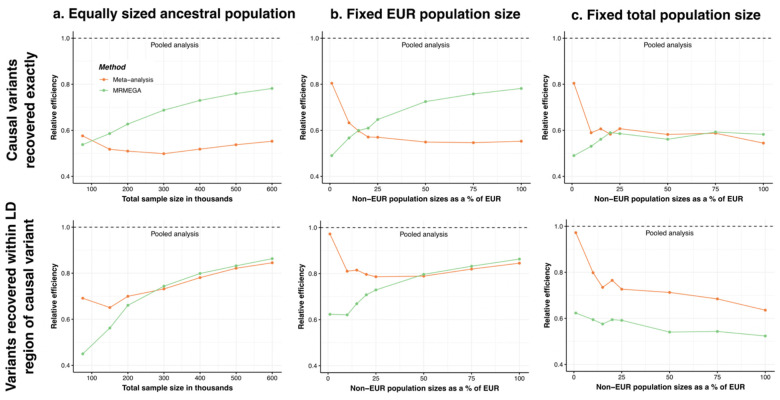
Comparison of meta-analysis and MR-MEGA with pooled analysis for detecting causal variants and variants in linkage disequilibrium (LD) across different ancestry-group proportions. This figure compares the relative efficiency of meta-analysis (orange) and MR-MEGA (green) in recovering causal variants and variants in LD, using pooled analysis (dashed line) as the benchmark (relative efficiency = 1.0). The top row (first panel) evaluates the exact recovery of causal variants, measured as the relative efficiency of identifying genome-wide significant ($P\leq 5\times 10^{-8}$) causal variants. The bottom row (second panel) assesses LD-based recovery of causal variants, which measures the relative efficiency of detecting causal variants that have at least one genome-wide significant variant within 500 kb. Each column represents a different study design: (a) equally sized ancestry groups (left), (b) a fixed EUR sample size of 120,000 with varying non-EUR sample sizes (middle), and (c) a fixed total sample size of 124,800 with varying non-EUR proportions (right). All methods are evaluated using GWAS summary statistics generated via REGENIE from simulations of continuous traits in a multi-ancestry dataset composed of African (AFR), Admixed American (AMR), East Asian (EAS), European (EUR), and South Asian (SAS) groups.

To assess statistical power across different ancestry-group proportions, we considered three scenarios: (1) equal sample sizes across ancestries while increasing total sample size, (2) a fixed EUR sample size while gradually increasing non-EUR sample size, and (3) a fixed total sample size with increasing non-EUR sample sizes. In all scenarios, pooled analysis consistently outperformed meta-analysis and MR-MEGA (Figure 4).

In the first scenario, where sample sizes were equal across ancestry groups (Figure 4a), meta-analysis initially outperformed MR-MEGA at smaller sample sizes, likely due to the additional parameter fitting required in MR-MEGA. However, once the total sample size exceeded 100,000 individuals (~20,000 per ancestry across five ancestry groups), MR-MEGA surpassed meta-analysis in identifying causal variants. For LD-based detection (i.e., identifying variants within 500 kb of a causal SNP), MR-MEGA outperformed meta-analysis when each ancestry group had at least 56,000 individuals (total n=280,000).

In the second scenario, we fixed at 120,000 individuals, while gradually increasing non-EUR sample sizes (Figure 4b). This setup reflects the impact of recruiting non-EUR participants within an existing EUR-dominated biobank. When non-EUR groups reached approximately 20% of the EUR sample size (i.e., 120,000 EUR and 24,000 of each non-EUR ancestry, total n=216,000), MR-MEGA outperformed meta-analysis in LD-based region detection. This advantages of MR-MEGA became more pronounced when non-EUR groups reached approximately 50% of the EUR sample size.

For the third scenario, we maintained a fixed total sample size of 124,800 individuals, while varying non-EUR proportions (Figure 4c). Here, meta-analysis consistently outperformed MR-MEGA for LD-based detection, regardless of ancestry-group proportions. However, MR-MEGA showed a slight advantage in identifying causal variants when all five ancestry-group were equally represented (24,960 per ancestry group). Pooled analysis exhibited the highest statistical power across all ancestry-group compositions particularly when ancestry-group proportions were more balanced. This advantage was most evident in LD-based region detection, where pooled analysis consistently outperformed both meta-analysis and MR-MEGA.

Across all scenarios, pooled analysis demonstrated the highest statistical power, regardless of ancestry-group proportions or total sample size. When only ancestry-group-specific summary statistics were available, MR-MEGA performed best when each ancestry group had at least 20,000 individuals, while meta-analysis remained preferable when some ancestry groups were smaller.

#### Binary Phenotypes

The results for binary phenotypes were consistent with those observed for continuous phenotypes (Table 1). Simulations were conducted using an average of 237,241 cases and 237,241 controls, with detailed sample sizes provided in Supplementary Table 3. Across all evaluated metrics, pooled analysis exhibited the highest statistical power. When measuring power by the proportion of causal variants reaching genome-wide significance ($P\leq 5\times 10^{-8}$) pooled analysis identified 0.33% of causal variants, compared to 0.17% for meta-analysis and 0.19% for MR-MEGA. Similarly, for LD-based detection, pooled analysis achieved a power of 27.19%, exceeding both meta-analysis (21.25%) and MR-MEGA (19.18%).

#### Admixed Individuals

To assess statistical power in samples including admixed individuals, we simulated four groups: two reference groups (100% AFR and 100% EUR) and two groups of admixed individuals (50%/50% AFR/EUR and 80%/20% AFR/EUR) using the Admix-kit^25^ pipeline, each with 60,000 subjects (Material and Methods). Across all groups, pooled analysis demonstrated the highest power for both detecting causal SNPs and identifying variants within LD-based causal regions (Table 2). For exact causal variant detection, pooled analysis achieved a power of 1.37%, outperforming both meta-analysis (0.83%) and MR-MEGA (0.91%). For LD-based detection, pooled analysis reached 29.93%, exceeding meta-analysis (24.31%) and MR-MEGA (23.56%).

### Real Data Analysis in AoU and UKB

We conducted GWAS using mixed-effect modelling with REGENIE and fixed-effect modelling with PLINK2 on real data from AoU and UKB, analyzing eight continuous and five binary traits (Materials and Methods). Sample sizes for each trait within ancestry groups are provided in Supplementary Tables 5–6, while Manhattan and Q-Q plots for all GWAS results are shown in Supplementary Figures 6–9. To assess inflation in test statistics, we examined the scaled genomic inflation factor ($\lambda_{1000}$), which adjusts for differences in sample size. Across all analyses, $\lambda_{1000}$ ranged from 0.999 to 1.003, indicating no or little systematic inflation was present. Since the true causal variants in real data are unknown, we measured statistical power by the number of genome-wide significant variants detected ($P\leq 5\times 10^{-8}$). Specifically, we reported both the total number of genome-wide significant variants and the number of independent genome-wide significant variants after LD-clumping.

Under both mixed- and fixed-effect modelling, pooled analysis consistently identified the highest number of significant variants, particularly for highly polygenic traits such as height. Across both AoU (Table 3, Supplementary Table 7) and UKB (Table 4, Supplementary Table 8), pooled analysis outperformed both meta-analysis and MR-MEGA for all traits, in both continuous and binary outcomes. On average, across all analysed traits in UKB, pooled analysis detected 8% and 60% more independent genome-wide significant variants than meta-analysis and MR-MEGA, respectively. The advantage of pooled analysis was even more pronounced in AoU, where it identified, on average across all traits, 63% and 650% more independent significant variants than meta-analysis and MR-MEGA, respectively. This greater advantage of pooled analysis in AoU is driven by its larger proportion of non-European participants (~41% non-EUR) compared to UKB (~4% non-EUR). This pattern aligns with our theoretical derivations (Materials and Methods), which predict that when MAFs vary across ancestries, pooled analysis has a greater advantage over meta-analysis when ancestry proportions are more balanced.

Additionally, mixed-effect models consistently outperformed fixed-effect models in detecting genome-wide significant variants. Across all three GWAS approaches, the mixed-effect model identified 6% more independent significant variants than the fixed-effect model in AoU (Table 3, Supplementary Table 7) and 18% more in UKB (Table 4, Supplementary Table 8). This increased statistical power of mixed-effect models is likely due to their ability to adjust for both known covariates (top PCs, age, and sex) through fixed effects and additional genomic structure through random effects. By modelling these sources of variation, mixed-effect models reduce residual error variance, thereby increasing statistical power.

## Discussion

We evaluated three multi-ancestry GWAS approaches, pooled analysis, meta-analysis, and MR-MEGA, under both fixed- and mixed-effect modelling. Our findings show that pooled analysis consistently provides the highest statistical power across various ancestry-group compositions and trait architectures, while maintaining well-controlled type I error in most realistic scenarios. This advantage is particularly pronounced when allele frequencies vary across ancestry groups, supporting our theoretical derivation that pooled analysis benefits from increased minor allele carrier counts across populations.

Through large-scale simulations, we showed that meta-analysis and MR-MEGA are effective but less powerful than pooled analysis, particularly when non-EUR individuals made up a larger proportion of the sample (Figure 4c). Our theoretical framework explains this disparity: as allele frequency divergence increases, meta-analysis becomes less efficient compared to pooled analysis. Real data analyses from UKB (n≈324,000) and AoU (n≈207,000) further validated our findings, with pooled analysis detecting the most independent genome-wide significant variants across both continuous and binary traits (Tables 3–4, Supplementary Tables 7–8).

Beyond allele frequency differences, sample size distribution across ancestry groups plays a key role in determining statistical power. When ancestry groups are more balanced, the power advantage of pooled analysis over meta-analysis and MR-MEGA is maximized. This is particularly evident in AoU, where a higher proportion of non-EUR participants amplifies the advantage of pooled analysis, further reinforcing our theoretical predictions.

Our findings also highlight the higher power of mixed-effect models over fixed-effect models (Tables 3–4, Supplementary Tables 7–8). By incorporating random effects, mixed-effect models account for genome-wide polygenic effects, which helps reduce residual error variance and improve the detection of true associations. Additionally, they adjust for cryptic relatedness and population structure, enhancing robustness while maintaining proper type I error control. These results support the continued adoption of efficient mixed-effect frameworks such as REGENIE for large-scale GWAS analyses.

Historically, fixed-effect meta-analysis has been the standard approach for multi-ancestry GWAS due to its ability to combine summary statistics from independent studies without requiring individual-level data^8,10,11,33,34^. However, our results suggest that pooled analysis is preferable when individual-level data are available, as it avoids binning individuals into discrete ancestry groups and leverages shared genetic architecture across populations. MR-MEGA, which models allele frequency differences, performed slightly better than standard meta-analysis when non-EUR sample sizes were sufficiently large, but it still lagged behind pooled analysis in most scenarios.

An important consideration is that pooled analysis inherently assumes homogeneous effect sizes across ancestry groups. While recent studies suggest that most genetic effects are shared across populations^35,36^, certain variants may exhibit ancestry-group-specific effect heterogeneity^37^, where meta-analysis might be more appropriate. Future methodological advancements, such as multi-trait^38^ or ancestry-aware fine-mapping approaches^39^, could refine pooled analysis to better capture ancestry-specific effects.

The observed power differences have important implications for PRS development and fine-mapping efforts in multi-ancestry cohorts. Pooled analysis may improve PRS portability by increasing statistical power for variant discovery, ultimately enhancing risk prediction models in different populations^40,41^. Additionally, the superior power of pooled analysis suggests that functional fine-mapping and transcriptome-wide association studies may benefit from using pooled GWAS summary statistics, as these analyses rely heavily on accurate effect size estimates^42^. Another key implication is for admixture mapping studies. Our findings indicate that pooled analysis provides better power than meta-analysis when analyzing admixed individuals, supporting its use in large, diverse cohorts such as AoU and the Million Veteran Program^43^. While meta-analysis may still be necessary when individual-level data are unavailable, future work should explore hybrid approaches that combine pooled analysis with ancestry-aware meta-analysis.

While our study provides strong evidence for the advantages of pooled analysis, there are important limitations. First, pooled analysis requires individual-level genotype data, which is often restricted due to privacy concerns and regulatory limitations. This constraint continues to favor meta-analysis for large-scale cross-cohort collaborations, highlighting the need for novel meta-analytic methods that retain the power of pooled approaches. Second, our simulations assume homogeneous allelic effects across ancestries, which may not fully reflect complex gene-environment interactions or ancestry-group-specific selection pressures. Future work should explore adaptive models that account for effect heterogeneity while maintaining the power benefits of pooled analysis. Third, while we demonstrated that pooled analysis maintains well-controlled type I error in most realistic settings, caution is needed when ancestry-group-specific PCs explain a large proportion (>1%) of trait variance, as this may lead to spurious associations. To mitigate this, we recommend evaluating the percentage of variance explained by ancestry-group-specific PCs prior to running pooled GWAS, particularly for traits with strong population structure effects.

In summary, our study demonstrates that pooled analysis provides the highest statistical power for multi-ancestry GWAS, particularly when allele frequencies vary across populations. While meta-analysis remains a valuable approach when individual-level data are unavailable, pooled analysis should be prioritized when feasible. Our findings emphasize the importance of balanced ancestry representation and highlight the advantages of mixed-effect modelling for large-scale genetic studies. Future research should focus on developing hybrid approaches that integrate the strengths of pooled and meta-analysis, ensuring that multi-ancestry GWAS continue to uncover novel genetic insights across diverse populations.

## Supplementary Material

Supplement 1

Supplement 2

## Figures and Tables

**Table 1:** Power comparison of multi-ancestry GWAS approaches on simulated binary phenotypes.

	No. samples on average	No. variants in GWAS output	No. significant variants	Power 1	Power 2
Pooled analysis	474,482	2,017,677	4,567	0.33%	27.19%
Meta-analysis	474,482	2,024,717	2,375	0.17%	21.25%
MR-MEGA	474,482	1,304,330	2,551	0.19%	19.18%

This table compares the performance of pooled analysis, meta-analysis and MR-MEGA, under mixed-effect modelling. The simulated dataset comprises of 237,241 cases and 237,241 controls from five ancestry groups: African (AFR), Admixed American (AMR), East Asian (EAS), European (EUR), and South Asian (SAS) populations. Detailed sample size by ancestry group is provided in Supplementary Table 3. The number of significant variants is determined using a genome-wide significance threshold of $P\leq 5\times 10^{-8}$. Power 1 represents the percentage of causal variants recovered as GWAS-significant variants. Power 2 represents the percentage of causal variants recovered within a 500kb region of GWAS-significant variants. The number of significant variants, Power 1, and Power 2 are averaged over 10 replicates. Further details on the simulation setup for phenotype generation can be found in the Material and Methods section.

**Table 2:** Power comparison of multi-ancestry GWAS approaches on simulated continuous phenotypes in equally sized samples of admixed individuals.

	No. samples	No. variants in GWAS output	No. significant variants	Power 1	Power 2
Pooled analysis	240,000	1,052,000	4,062	1.37%	29.93%
Meta-analysis	240,000	1,052,000	2,633	0.83%	24.31%
MR-MEGA	240,000	1,011,016	3,044	0.91%	23.56%

This table compares the performance of three multi-ancestry GWAS approaches: pooled analysis, meta-analysis and MR-MEGA, under mixed-effect modelling. The dataset comprises of one set of 60,000 samples simulated based on 1000G European (EUR) genotypes and one set of 60,000 samples simulated based on 1000G African (AFR) genotypes, one set of 60,000 individuals with 50/50 EUR/AFR admixture, and one set of 60,000 individuals with 20/80 EUR/AFR admixture. Simulations were performed using Admix-kit^25^. The number of significant variants was determined using a genome-wide significance threshold of $P\leq 5\times 10^{-8}$. Power 1 represents the percentage of causal variants recovered as GWAS-significant variants. Power 2 represents the percentage of causal variants recovered within a 500kb of GWAS-significant variants. The number of significant variants, Power 1, and Power 2 are averaged over ten replicates. Further details on the simulation setup for phenotype generation can be found in the Material and Methods section.

**Table 3:** Comparison of significant and independent variants across multi-ancestry GWAS methods in All of Us with mixed-effect modelling.

		Height	Waist	LDL	TC	HDL	Calcium	Creatinine	eGFR	Asthma	CAD	T2D	Breast cancer	Prostate cancer
Number of significant variants	Pooled analysis	5,435	2,120	354	921	1,258	73	513	37	101	125	268	20	35
	Meta-analysis	4,668	823	339	550	760	62	73	11	99	124	287	20	35
	MR-MEGA	3,695	627	303	475	628	31	25	3	40	120	280	20	22
Number of independent significant variants	Pooled analysis	580	316	42	50	101	18	213	1	7	3	28	2	11
	Meta-analysis	559	80	36	39	87	15	43	1	7	3	24	2	8
	MR-MEGA	394	44	22	27	52	6	3	1	4	2	16	2	5

This table compares the number of significant and independent variants identified using three different multi-ancestry GWAS approaches: pooled analysis, meta-analysis and MR-MEGA. Variants were filtered based on a minor allele frequency (MAF) > 0.01 in at least one ancestry group, resulting in approximately 1.2 million variants analyzed. Significant variants were determined using a genome-wide significance threshold of $P\leq 5\times 10^{-8}$. Independent variants are generated with linkage disequilibrium (LD) clumping with an ${\text{r}}^{2}$ threshold of 0.1. The eight continuous phenotypes include height, waist circumference, low-density lipoproteins cholesterol (LDL), total cholesterol (TC), high-density lipoproteins cholesterol (HDL), calcium, creatinine, estimated glomerular filtration rate (eGFR). The five binary phenotypes include asthma, coronary artery disease (CAD), type II diabetes (T2D), breast cancer and prostate cancer. The number of subjects varies by phenotype, and the sample size for each trait is provided in Supplementary Table 5.

**Table 4:** Comparison of significant and independent variants across multi-ancestry GWAS methods in the UK Biobank with mixed-effect modelling.

		Height	Waist	LDL	TC	HDL	Calcium	Creatinine	eGFR	Asthma	CAD	T2D	Breast cancer	Prostate cancer
Number of significant variants	Pooled analysis	44,509	5,393	6,580	7,903	10,132	3,758	3,582	14,174	1,010	752	742	207	205
	Meta-analysis	45,001	5,196	6,532	7,792	9,624	3,750	3,664	12,738	1,039	675	745	211	186
	MR-MEGA	33,844	3,629	4,743	5,884	6,871	2,690	2,578	9,054	791	453	509	135	125
Number of independent significant variants	Pooled analysis	2,576	351	345	401	604	271	378	743	37	43	52	15	28
	Meta-analysis	2,574	338	344	399	595	270	232	731	34	41	53	14	25
	MR-MEGA	1,851	224	232	266	404	184	164	505	26	24	32	11	15

This table compares the number of significant and independent variants identified using three multi-ancestry GWAS approaches: pooled analysis, meta-analysis and MR-MEGA. Variants were filtered using a MAF threshold of > 0.01 within at least one ancestry group, resulting in approximately 1.53 million variants analyzed. Significant variants were determined using a genome-wide significance threshold of $P\leq 5\times 10^{-8}$. Independent variants are generated with linkage disequilibrium (LD) clumping with an $r^{2}$ threshold of 0.1. The eight continuous phenotypes include height, waist circumference, low-density lipoproteins cholesterol (LDL), total cholesterol (TC), high-density lipoproteins cholesterol (HDL), calcium, creatinine, estimated glomerular filtration rate (eGFR). The five binary phenotypes include asthma, coronary artery disease (CAD), type II diabetes (T2D), breast cancer and prostate cancer. The number of subjects varies by phenotype, and sample sizes for each trait are provided in Supplementary Table 6.

## Data Availability

The simulated data for 600,000 subjects from five ancestries were downloaded from here: https://dataverse.harvard.edu/dataset.xhtml?persistentld=doi:10.7910/DVN/COXHAP. The UK Biobank phenotype and genotype data used in this study are available to registered researchers through the UKB data-access protocol and require an approved access application. All of Us phenotype and genotype data can be accessed through the All of Us Research Workbench (https://workbench.researchallofus.org/). All data used in this study are available to registered researchers with controlled-tier access through the All of Us data-access protocol.
